# ICP Versus Laser Doppler Cerebrovascular Reactivity Indices to Assess Brain Autoregulatory Capacity

**DOI:** 10.1007/s12028-017-0472-x

**Published:** 2017-10-17

**Authors:** F. A. Zeiler, J. Donnelly, D. Cardim, D. K. Menon, P. Smielewski, M. Czosnyka

**Affiliations:** 1 Division of Anaesthesia, Addenbrooke’s Hospital, University of Cambridge, Cambridge, UK; 2 Section of Surgery, Rady Faculty of Health Sciences, University of Manitoba, Winnipeg, MB Canada; 3 Clinician Investigator Program, Rady Faculty of Health Science, University of Manitoba, Winnipeg, MB Canada; 4 Section of Brain Physics, Division of Neurosurgery, Department of Clinical Neurosciences, Addenbrooke’s Hospital, University of Cambridge, Cambridge, CB2 0QQ UK; 5 Neurosciences Critical Care Unit, Addenbrooke’s Hospital, Cambridge, UK; 6 Queens’ College, University of Cambridge, Cambridge, UK; 7 National Institute for Health Research, Cambridge, UK; 8 Institute of Electronic Systems, Warsaw University of Technology, Warszawa, Poland

**Keywords:** Cerebrovascular reactivity, Autoregulation, Laser Doppler, ICP index, Covariance, Machine learning

## Abstract

**Background:**

To explore the relationship between various autoregulatory indices in order to determine which approximate small vessel/microvascular (MV) autoregulatory capacity most accurately.

**Methods:**

Utilizing a retrospective cohort of traumatic brain injury patients (*N* = 41) with: transcranial Doppler (TCD), intracranial pressure (ICP) and cortical laser Doppler flowmetry (LDF), we calculated various continuous indices of autoregulation and cerebrovascular responsiveness: A. ICP derived [pressure reactivity index (PRx)—correlation between ICP and mean arterial pressure (MAP), PAx—correlation between pulse amplitude of ICP (AMP) and MAP, RAC—correlation between AMP and cerebral perfusion pressure (CPP)], B. TCD derived (Mx—correlation between mean flow velocity (FVm) and CPP, Mx_a—correlation between FVm and MAP, Sx—correlation between systolic flow velocity (FVs) and CPP, Sx_a—correlation between FVs and MAP, Dx—correlation between diastolic flow index (FVd) and CPP, Dx_a—correlation between FVd and MAP], and LDF derived (Lx—correlation between LDF cerebral blood flow [CBF] and CPP, Lx_a—correlation between LDF-CBF and MAP). We assessed the relationship between these indices via Pearson correlation, Friedman test, principal component analysis (PCA), agglomerative hierarchal clustering (AHC), and *k*-means cluster analysis (KMCA).

**Results:**

LDF-based autoregulatory index (Lx) was most associated with TCD-based Mx/Mx_a and Dx/Dx_a across Pearson correlation, PCA, AHC, and KMCA. Lx was only remotely associated with ICP-based indices (PRx, PAx, RAC). TCD-based Sx/Sx_a was more closely associated with ICP-derived PRx, PAx and RAC. This indicates that vascular-derived indices of autoregulatory capacity (i.e., TCD and LDF based) covary, with Sx/Sx_a being the exception, whereas indices of cerebrovascular reactivity derived from pulsatile CBV (i.e., ICP indices) appear to not be closely related to those of vascular origin.

**Conclusions:**

Transcranial Doppler Mx is the most closely associated with LDF-based Lx/Lx_a. Both Sx/Sx-a and the ICP-derived indices appear to be dissociated with LDF-based cerebrovascular reactivity, leaving Mx/Mx-a as a better surrogate for the assessment of cortical small vessel/MV cerebrovascular reactivity. Sx/Sx_a cocluster/covary with ICP-derived indices, as seen in our previous work.

**Electronic supplementary material:**

The online version of this article (doi:10.1007/s12028-017-0472-x) contains supplementary material, which is available to authorized users.

## Introduction

Continuous assessments of autoregulation/cerebrovascular reactivity in traumatic brain injury (TBI) patients focus on the calculation of moving Pearson correlation coefficients between physiologic variables that characterize systemic and cerebrovascular dynamics [1–3]. These indices are derived by comparing slow wave changes of a surrogate for cerebral blood volume (CBV)/cerebral blood flow (CBF), to the intravascular driving force, mean arterial pressure (MAP) or cerebral perfusion pressure (CPP) [1, 3]. Commonly monitored surrogates for slow waves of CBV and CBF are intracranial pressure (ICP) and transcranial Doppler (TCD)-based CBF velocity (CBFV). The correlation coefficient between various combinations of these physiologic variables carries information related to the phase shift between these signals [3, 4]. Positive and negative correlation coefficients typically denote “impaired” and “intact” autoregulatory capacity/cerebrovascular reactivity, respectively.

Pressure reactivity index (PRx), derived from ICP and MAP, and mean flow index (Mx), derived from transcranial Doppler-derived CBFV and CPP, are the two most commonly quoted continuous indices of cerebrovascular reactivity in TBI. Critical thresholds for both morbidity and mortality exist for both PRx [5] and Mx [6], with moderate inter-index correlation (*r* values quoted up to 0.58) [7]. However, other continuous indices of autoregulation/cerebrovascular reactivity, using multimodal monitoring, have also been employed [3], with variable levels of validation. Given the different monitoring techniques utilized to produce these indices, these carry different physiologic information, and may not provide similar information regarding cerebral autoregulation/vessel reactivity.

Though no longer employed clinically, laser Doppler flowmetry (LDF) affords the ability to obtain continuous direct measure of small vessel/microvascular (MV) CBF [8–11]. This device requires insertion into the subdural space and uses the Doppler shift in the reflected light signal to calculate cortical CBF in the region of the probe [8]. Given the availability of this direct measure of cerebral MV flow, it is worth asking how Mx and other TCD-derived indices of cerebrovascular reactivity (which are indices based on regional CBF velocity in a single vascular territory—typically the middle cerebral artery [MCA]), relate to MV autoregulatory capacity.

A previous study [12] showed interesting differences between Lx (correlation between LDF-CBF and CPP) and Mx, but did not address relationships with other TCD-based indices (such as Sx—correlation between systolic flow velocity and CPP, and Dx—correlation between diastolic flow velocity and CPP). Further, the existing data provide no guidance on how PRx (and other ICP-derived indices of cerebrovascular reactivity based on “global” ICP), relate to MV autoregulatory capacity. While focal continuous assessment MV CBF is possible with thermal diffusion catheters, their use in the assessment of MV behavior over extended periods is limited, given the need for repeated re-calibration and moderate noise in the parent signal derived [12]. However, given that, of all the continuous bedside monitors available, the MV flow best approximates nutritive perfusion, such relationships are critically important in helping validate and interpret less direct metrics of vascular biology in the injured brain.

The goal of this retrospective cohort study is to explore the relationship between various commonly used bedside autoregulatory/cerebrovascular reactivity indices in order to determine which indices best approximate cortical small vessel/MV autoregulatory capacity. We employ various tests of multivariate assessment of covariance in order to assess these relationships, similar to a recent publication from our group on covariance/clustering of multimodal monitoring-based continuous autoregulation/cerebrovascular reactivity indices [13].

## Methods

### Patient Population

The patient population included in this study is a subpopulation of a cohort that has been previously described [8, 11]. This patient cohort was one in which the main goal of the initial prospective study was to assess regional CBF via LDF in TBI patients, where local Cambridge Health Authority research ethics committee approval was obtained. Through retrospective analysis of this cohort, we identified that the raw monitoring signals included data that would allow us to determine various indices of autoregulatory capacity/cerebrovascular reactivity, assessing the relationship between those derived from different monitoring devices. All recording sessions included in this study had the following monitors: ICP, MAP, CPP, LDF-CBF, and TCD-based CBFV of the middle cerebral artery (MCA) ipsilateral to the ICP and LDF monitors.

This study was conducted as a retrospective analysis of a prospectively maintained database cohort, in which 61 separate recordings were analyzed. Most recordings were approximately 30 min–1 h in duration. All patients in both cohorts suffered moderate–severe TBI, or deteriorated after an initial admission with mild TBI and required sedation and mechanical ventilation for clinical care in the Neurosciences Critical Care Unit at Addenbrooke’s Hospital, Cambridge. Treatment received during the recording periods included standard ICP-directed therapy, with an ICP goal of less than 20 mm Hg and CPP goal of greater than 60 mm Hg. All patients were nursed with head of the bed at 30°. For refractory elevations in ICP, bolus dosing of mannitol was administered (for ICP > 25 mm Hg for 15 min). From the available records, no patients underwent therapeutic hypothermia therapy for refractory ICP.

Data on age, injury severity, and clinical status at hospital discharge were recorded at the time of monitoring on this database, and no attempt was made to re-access clinical records for additional information. Since all data were extracted from the hospital records and fully anonymized, no data on patient identifiers were available, and formal patient or proxy consent was not sought.

### Signal Acquisition

Various signals were obtained through a combination of invasive and noninvasive methods. Arterial blood pressure (ABP) was obtained through either radial or femoral arterial lines connected to pressure transducers (Baxter Healthcare Corp. CardioVascular Group, Irvine, CA). ICP was acquired via an intraparenchymal strain gauge probe (Codman ICP MicroSensor; Codman & Shurtleff Inc., Raynham, MA).

LDF-based CBF was obtained via placement of a MBF3D dual-channel laser LDF (Moor Instrument Ltd, Devon UK) in the subdual space, ipsilateral to the ICP monitor. The LDF probe employed a low energy laser (0.5–1.5 mW) with light generated in the near-infrared spectrum (780–820 nm). LDF signals were recorded at a frequency of 14.6 kHz. All probes were precalibrated prior to insertion. Details on the insertion technique and calibration method can be found in the 1994 study by Kirkpatrick et al. [8].

Finally, TCD assessment of MCA CBFV was conducted via Doppler Box (DWL Compumedics, Singen, Germany) or Neuroguard (Medasonic, Fremont, CA, USA). Unilateral MCA recordings (ipsilateral to the ICP and LDF monitors) were obtained in every patient during these sessions.

All recorded signals were digitized via an A/D converters (DT9801; Data Translation, Marlboro, MA), sampled at frequency of 50 Hertz (Hz) or higher and recorded using WREC software (Warsaw University of Technology) and analyzed retrospectively using ICM+ software (Cambridge Enterprise Ltd, Cambridge, UK, http://www.neurosurg.cam.ac.uk/icmplus). All signal artifacts were removed prior to further processing or analysis.

### Signal Processing

Post-acquisition processing of the above described signals was conducted utilizing ICM+ software. CPP was determined utilizing the virtual signals by: CPP = MAP—ICP. Systolic ABP (ABPs) was determined by calculating the maximum ABP over a 1.5 s window, updated every second. Similarly, diastolic ABP (ABPd) was also determined by calculating the minimum ABP over a 1.5 s window, updated every second. Systolic flow velocity (FVs) was determined by calculating the maximum flow velocity (FV) over a 1.5 s window, updated every second. Diastolic flow velocity (FVd) was calculated using the minimum FV over a 1.5 s window, updated every second. Mean flow velocity (FVm) was calculated using average FV over a 10 s window, updated every 10 s (i.e., without data overlap). Pulse amplitude of ICP (AMP) was determined by calculating the fundamental amplitude of the ICP signal over a 10 s window, updated every 10 s. Ten second moving averages (updated every 10 s to avoid data overlap) were calculated for all recorded signals: ICP, ABP (which produced MAP), ABPs, ABPd, CPP, FVm, FVs, FVd, and LDF-CBF.

### Autoregulation/Cerebrovascular Reactivity Indices

The autoregulation/cerebrovascular reactivity indices were derived in a similar fashion, the example provided is for PRx. A moving Pearson correlation coefficient was calculated between ICP and MAP using 30 consecutive 10 s windows (i.e., five minutes of data), updated every 10 s. A 10 s update period was chosen given the short duration of the recordings. Details on each index calculation can be found in Appendix A of the supplementary materials.

### Statistics

The analysis conducted is identical to that performed in our previous publication on covariance of multimodal monitoring autoregulation/cerebrovascular reactivity indices [13]. The only difference for this study is that we have slightly larger patient/recording numbers, no brain tissue oxygenation or near infrared spectroscopy (NIRS) monitoring, and the presence of LDF signal.

### General Statistics

Data were provided on a 10 s-to-10 s basis for the duration of the recordings for each recording. This was extracted from ICM+ in to comma separated variable (CSV) documents, which were collated into one continuous data sheet (compiled from all patients). We then determined individual recording grand means for each variable. The statistical analysis was performed on both data sheets: 10 s-by-10 s data and grand mean data.

Statistics were performed utilizing XLSTAT (Addinsoft, New York, United States; https://www.xlstat.com/en/) add-on package to Microsoft Excel (Microsoft Office 15, Version 16.0.7369.1323) and R statistical software [14]. Tests for normality were performed using the Shapiro–Wilks test for all indices and measured variables. All indices and variables were determined to be nonparametric in nature. Alpha was set at 0.05 for all results describing a *p* value.

### Autoregulation/Cerebrovascular Index: Correlative Statistics

For assessment of the autoregulatory indices, we employed a Pearson correlation coefficient matrix to assess correlation between the various indices, which was conducted after performing a Fisher transformation to the data set (given nonparametric distribution for each index). This was the only test in which transformed data were utilized within the analysis.

Grouped variance between different combinations of indices was assessed using the Friedman test (with and without multiple comparisons), to account for within subject variation. The main assumption made was that all indices were measuring the same physiologic variable (i.e., autoregulation). The Friedman test was performed on the following groups: all indices, ICP-derived indices (PRx, PAx, RAC), TCD-derived indices (Mx, Sx, Dx, Mx_a, Sx_a, Dx_a) and LDF-CBF-derived indices (Lx, Lx_a). Given the results of the Friedman test were similar for both with and without multiple comparisons, we only mention the “with” multiple comparisons data within the manuscript and supplementary material.

### Multivariate Clustering and Assessment of Covariance

Finally, multivariate statistics were performed to further delineate the associations between the various indices. Currently, it is unclear as to which multivariate clustering technique is superior within the exploration of time series-based physiologic variables, thus we chose to employ an array of testing techniques. Three different multivariate methods were employed in order to assess the covariance within various combinations of indices. This was done, so as to be comprehensive and to provide confirmation of the potential clustering seen in any individual given test. This analysis was identical to that performed in our previous publication, assessing covariance and clustering between numerous monitoring-based continuous indices of cerebrovascular reactivity [13].

First, principal component analysis (PCA) was performed using a Spearman-type PCA, chosen to account for the nonparametric data distribution in the data set (with significance set at *p* < 0.05). The PCA has been described in detail in other publications and is ideally suited as an “exploratory” statistic for small patient cohorts with large numbers of variables [15, 16]. The purpose of the PCA is to highlight which combinations of variables explain the overall variance within the entire data set, and thus which variables may be related and of further interest to study via other methods. We refer the readers to cited publications on PCA for more information [15, 16].

Second and third, agglomerative hierarchal clustering (AHC) and *k*-mean-based cluster analysis (KMCA) (using Euclidean distance) were also performed. These tests provide an overall assessment of the similarity between variables, grouping them into clusters (or stems on a dendrogram, as seen within AHC) based on the mean distance away from one another, as assessed by Euclidean distance.

For the AHC, the statistical strength of the correlation between the clusters produced in the dendrograms was quantified using cophenetic correlation coefficients [17]. Cophenetic correlation coefficients were produced by the Spearman correlation between the original Euclidean distance matrix calculated for the AHC, and the cophenetic distance matrix. The cophenetic distance is defined as the distance between two clusters that contain two indices individually and the point where both clusters are merged (i.e., it represents the height on the dendrogram at which the branch points occur). The cophenetic correlation coefficient is believed to be an estimate of how well the AHC dendrogram maintains pairwise distances when compared with the original data set (i.e., the baseline distance matrix between variables).

With the KMCA, the number of clusters can be set by the investigator. We utilized the “Elbow method” of KMCA in order to determine the appropriate number of clusters for the final analysis. The Elbow method consists of computing all possible *k*-means clusters. Subsequently, a plot of the within-group sum of squares versus cluster number, allowed selection of an inflection point (or “elbow”) at which the plot showed the most dramatic slope change. This is deemed the “most appropriate” cluster number for the final analysis.

## Results

### Patient Demographics

**Fig. 1:**
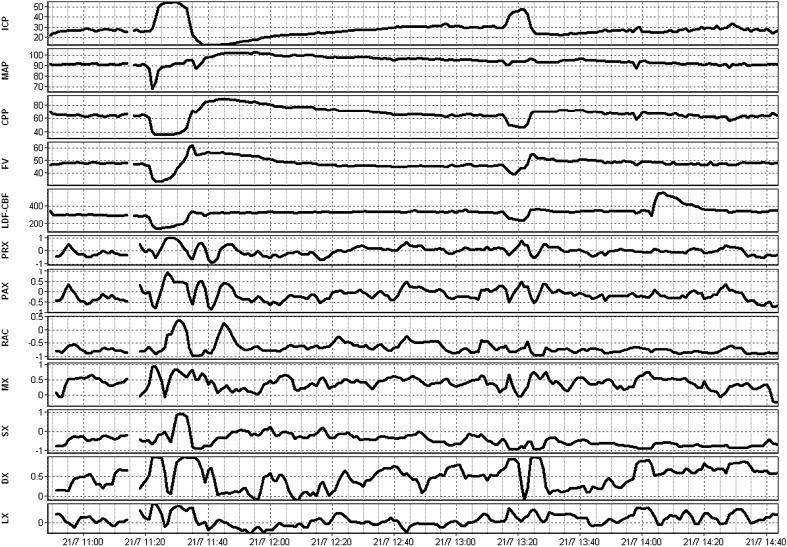
Example of parent signal and autoregulation index fluctuations during plateau wave. *AMP* fundamental amplitude of ICP, *a.u*. arbitrary units, *CPP* cerebral perfusion pressure, *Dx* diastolic flow index (between FVd and CPP), *FVd* diastolic flow velocity, *FVm* mean flow velocity, *FVs* systolic flow velocity, *ICP* intracranial pressure, *LDF* laser Doppler flowmetry, *LDF-CBF* LDF cerebral blood flow, *Lx* laser Doppler flow index (between LDF-CBF and CPP), *Mx* mean flow index (between FVm and CPP), *PAx* between AMP and MAP, *PRx* pressure reactivity index (between ICP and MAP), *RAC* between AMP and CPP, *TCD* transcranial Doppler. ICP, MAP, and CPP are measured in mm Hg. LDF-CBF, PRx, PAx, RAC, Mx, Sx, Dx, and Lx are all measured in a.u

A total of 40 patients, with 61 recordings, were included within this study. The average age was 31.1 ± 15.3 years, with a median admission Glasgow Coma Scale of 5 (IQR 4–7). The median 6-month Glasgow Outcome Score for these patients was 2 (range 1–5). Given the age of the data (i.e., early 1990’s) the available information is limited for patient demographics and injury characteristics. No archived imaging information was available to allow us to determine injury patterns. Further clinical details on subpopulations of this cohort can be found in other publications on this cohort [8, 11]. Figure 1 displays an example of signal and autoregulatory/cerebrovascular reactivity index responsiveness during a plateau wave in a patient with LDF monitoring.

### Autoregulation/Cerebrovascular Reactivity Index Analysis

#### Inter-index Correlation

We compared the inter-index correlation via a Pearson correlation matrix, for both the 10 s-by-10 s data and the grand mean data. The Pearson matrices, with *p* value matrices, for both data sheets can be found in Appendix B of the Supplementary materials. Of note, the ICP-derived indices (PRx, PAx and RAC) display moderate-to-strong inter-technique correlation (*r* ≳ 0.5 in all, *p* < 0.05 in all). A similar trend was noted with the TCD-derived indices (Mx, Mx_a, Dx, Dx_a, Sx, Sx_a). Mx and PRx were correlated (*r* = 0.346, *p* = 0.006). Sx and Sx_a were moderately correlated with the ICP-derived indices. Finally, the LDF-derived indices were correlated more with TCD indices (Mx: *r* = 0.561, *p* < 0.0001; Dx: *r* = 0.492, *p* < 0.0001). Thus, it appears that cortical small vessel/MV autoregulatory capacity may be better approximated by TCD-derived Mx/Mx_a and Dx/Dx_a, versus other indices. These relationships were confirmed in both data sheets.

#### Grouped Variance Analysis: Friedman Test

Similarity between various groups of autoregulatory indices was assessed by the Friedman test (with and without multiple comparisons), with the pretest assumption that each index was assessing the same aspect of physiology, autoregulation. In both the 10 s-by-10 s and grand mean data sheets, Friedman testing confirmed that the indices were not all the same (*p* < 0.0001, *Q* = 301.204). Further Friedman tests were applied to groups of monitor-specific indices (i.e., derived indices were grouped based on their monitoring signal source: ICP, TCD, etc). The within monitor Friedman testing also confirmed each index was in fact different. A summary of the Friedman test results (with multiple comparisons) for both data sheets can be seen in Appendix C of the Supplementary Materials.

#### Principle Component Analysis

Spearman PCA was conducted on both data sheets, with similar results. Eleven principal components (PC) (also referred to as factors [F]) were identified, with the first 5 PC’s composing ~90% of the overall variance in the data set. PC eigenvalue data, scree plots, and variable-specific loadings can be seen in Appendix D of the supplementary materials.

A loading biplot for PC1 (denoted F1) and PC2 (denoted F2) can be seen in Appendix C. As can be seen within the biplot, the ICP-derived indices (PRx, PAx and RAC) are clustered in the same quadrant of the biplot, contributing to the overall variance of both PC1 and PC2. Furthermore, PRx/PAx/RAC appeared to be associated with TCD-based Sx and Sx_a, in terms of their contributions to the variance of the whole data set. Similarly, the TCD-based indices (Mx, Mx_a, Dx and Dx_a) were colocated within the area of the biplot most associated with PC1. LDF indices (Lx, Lx_a) covaried with TCD-derived Mx/Mx_a/Dx/Dx_a, confirming the correlations seen in the Pearson analysis.

#### Agglomerative Hierarchal Clustering

**Fig. 2:**
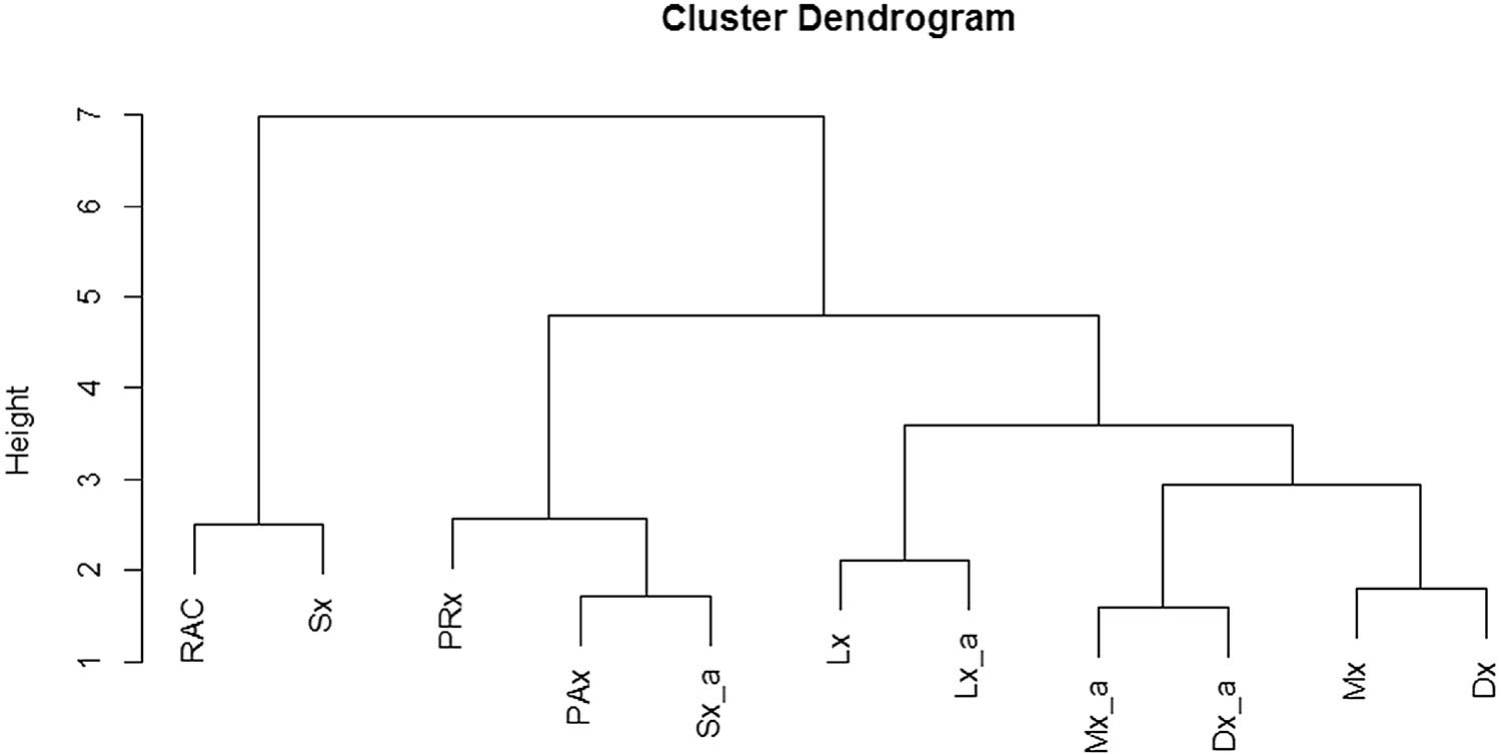
AHC of autoregulatory indices—grand mean data. *AHC* agglomerative hierarchal clustering, *AMP* fundamental amplitude of ICP, *CPP* cerebral perfusion pressure, *Dx* diastolic flow index (between FVd and CPP), *Dx_a* arterial diastolic flow index (between FVd and MAP), *FVd* diastolic flow velocity, *FVm* mean flow velocity, *FVs* systolic flow velocity, *ICP* intracranial pressure, *Lx* laser Doppler flow index (between LDF-CBF and CPP), *Lx_a* arterial laser Doppler flow index (between LDF-CBF and MAP), *Mx* mean flow index (between FVm and CPP), *Mx_a* arterial mean flow index (between FVm and MAP), *PAx* between AMP and MAP, *PRx* pressure reactivity index (between ICP and MAP), *RAC* between AMP and CPP

AHC was performed on both data sheets, yielding identical results. Figure 2 demonstrates the dendrogram produced. Of note is the clustering of ICP, TCD and LDF-based indices. ICP indices cocluster with Sx and Sx_a, as displayed in both Pearson and PCA testing. Similarly, TCD-based Mx/Mx_a and Dx/Dx_a cocluster with Lx/Lx_a, as seen in the Pearson and PCA testing. The cophenetic correlation coefficient for the grand mean AHC was 0.77, indicating moderate-to-strong significance of the clustering. The dendrogram for the 10 s-by-10 s data can be seen in Appendix E of the Supplementary Materials.

#### *K*-Means Cluster Analysis

KMCA was performed on both data sheets, producing identical clustering results. Based on the “Elbow Method,” the optimal number of centroids for the KMCA was determined to be 4. The clustering of the indices was similar to that seen in the AHC, PCA and Pearson testing. Details of the KMCA and cluster tables can be seen in Appendix F of the Supplementary Materials.

## Discussion

Through the analysis of this TBI LDF data set, we have been able to better define the relationships between various ICP/TCD/LDF autoregulatory indices in humans. First, intratechnique correlations were seen for ICP, TCD, and LDF-CBF-derived indices across Pearson, PCA, ACH, and KMCA. This result is not surprising, given indices derived from the same signals, should be expected to be inter-related. Second, LDF-CBF-based Lx and Lx_a were found to be more closely associated with TCD-based Mx/Mx_a and Dx/Dx_a, than with Sx/Sx_a or the ICP-derived indices. This was confirmed on all forms of the analysis. This suggests that TCD “vascular”-based measures (Mx/Mx_a and Dx/Dx_a) are a better approximation of cortical small vessel/microcirculatory autoregulation. Third, Sx/Sx_a appear to be more closely associated with the ICP-derived indices (PRx, PAx and RAC), as confirmed on all forms of the analysis. This was also seen in our previously published work [13]. This likely stems from the peak pulsatile systolic component of CBFV yielding a stronger contribution to the ICP signal, than mean or diastolic CBFV’s. Further, it is not surprising that by the time that CBF reaches the small cortical vessels that the peak systolic pulsatile component has less of an impact on regional LDF-CBF signal, where it is more likely to be dependent on mean flow or diastolic flow parameters. This, however, requires confirmation.

### Limitations

Some important limitations should be highlighted. First, this is a small retrospective cohort of patients that were studied. The patients had heterogeneous injury patterns and were subject to variations in intensive care unit therapies/treatments during the short recording sessions. This could have impacted signal heterogeneity and quality, leading to a direct influence on both the results of the slow wave and autoregulatory index analysis. Further to this, given the age of the initial data (ie. early 1990’s), we were limited in the available patient demographics and intracranial injury pattern/burden. Limited paper records were available and no archived imaging was available. As a result, we cannot comment on the impact of various patient comorbid factors or injury pattern/burden factors on the various autoregulatory indices. In addition, these patients were not randomized in any fashion, but were merely a unique cohort with ICP, LDF, and TCD high frequency signal linked in time series, allowing for an interesting retrospective analysis. Therefore, the strength of conclusions that can be drawn from our analysis is limited. However, with that said, we do believe the analysis conducted provides more than anecdotal insight into the covariance and inter-index relationship, providing valuable information all involved in the critical care management of moderate/severe TBI patients. Second, LDF-CBF probes are no longer in clinical use in humans, thus despite the interesting trends, it proves difficult to confirm the analysis with newer and larger patient cohorts. Therefore, we are unfortunately left with retrospective data sets like these or animal studies still employing LDF, to analyze relationships of still commonly applied monitors to LDF-based cortical/small vessel CBF. The decline in their use stemmed from cost, maintenance, invasive placement, and focality of measure. Not to mention the relatively noisy signal generated from red blood cell flux measurements. With that said, they provided useful and unique information on cortical cerebral blood flow, and subsequent cerebrovascular reactivity. Currently, the spatially resolved NIRS-based continuous autoregulatory index TOx (also known as COx) is the only index, aside from PRx, that has been validated in an animal model against the lower limit of autoregulation, with LDF providing the continuous assessment of CBF during this study [18]. Thus, this TOx index, the correlation between total oxygenation index and CPP, may be the closest surrogate to LDF-based indices. However, this has not been proven, as we are unaware of any human-based data set of high frequency time series including ICP, LDF and NIRS monitoring. A potential solution, though not necessarily definitive for clinical application, would be a similar covariance analysis conducted in this previously described animal data set [18]. Third, the statistics utilized within the autoregulatory index analysis are mainly exploratory and not 100% confirmatory of the relationships described. The use of PCA, AHC, and KMCA is exploratory multivariate statistical techniques designed to highlight potential relationships of interest within an entire data set, which would then drive further prospective focused assessment of the individual relationships identified. Given the limitations mentioned around the clinical use of LDF, the analysis will have to remain “exploratory” for human data. With that said, the relationships were all confirmed across Pearson, PCA, AHC, and KMCA, potentially indicating that the various clustering/correlations are more than just by chance within an individual multivariate test. In addition to this, future prospective evaluation of the index relations can be carried out within controlled animal studies, given the continued application of LDF within this setting. Finally, the use of the Friedman test within the context of comparing various indices derived from various monitoring devices is controversial. We made the assumption that all indices were measuring the same biological construct—autoregulatory capacity. The results of this analysis of variance between indices should be interpreted with caution.

### Future Considerations

Based on the current available literature on cerebral autoregulation in humans, it is unknown as to what defines the “gold standard” for cortical microcirculatory autoregulatory capacity. The most commonly employed index of autoregulation is PRx, a variable derived from a global ICP measure, and based on our work above, does not appear closely associated with cortical indices. LDF had the potential to define cortical pial/microcirculatory reactivity, though has fallen out of favor, leaving only these small unique data sets to provide limited insight into cortical autoregulation.

These devices are still employed in animal studies, and this may provide the next logical avenue for comparison of existing, commonly employed, monitoring devices and the continuous indices of cerebrovascular reactivity derived from their signals. Through comparing current, and emerging, multimodal monitoring-based continuous assessments of cerebrovascular reactivity to LDF-based indices in animal models, we may be able to more accurately characterize which monitoring variables are linked to cortical autoregulatory capacity, and potentially provide a “surrogate” measurement technique for LDF in humans.

It has yet to fully uncovered in the literature, but knowledge of cortical microcirculatory autoregulatory capacity may prove to be dramatically different than the existing global-based assessment (ie. PRx). It may be that impairment of cortical cerebrovascular reactivity has more of a relationship to global outcome measures, or even more subtle executive functioning capabilities in the long-term post-TBI, or other cerebral insult. Additional work is required.

## Conclusions

Of the bedside indices of autoregulation in common use, TCD-based metrics, and Mx in particular, are most closely related to LDF-derived measures of MV flow (Lx/Lx_a). Both Sx/Sx-a and the ICP-derived indices appear to be dissociated from LDF-based cortical small vessel cerebrovascular reactivity, leaving Mx/Mx-a/Dx/Dx-a as a better surrogate for the assessment of cortical small vessel/MV cerebrovascular reactivity. Sx/Sx_a cocluster/covary with ICP-derived indices, as seen in our previous work.

## Electronic supplementary material

Below is the link to the electronic supplementary material.

Supplementary material 1 (DOCX 13 kb)

Supplementary material 2 (DOCX 34 kb)

Supplementary material 3 (DOCX 13 kb)

Supplementary material 4 (DOCX 189 kb)

Supplementary material 5 (DOCX 45 kb)

Supplementary material 6 (DOCX 94 kb)
