# Supplementary material for: ICP Versus Laser Doppler Cerebrovascular Reactivity Indices to Assess Brain Autoregulatory Capacity
Source: Neurocrit Care. 2017 Oct 17;28(2):194–202. doi: 10.1007/s12028-017-0472-x (PMC5948245; doi:10.1007/s12028-017-0472-x)
Supplement: Supplementary file 1 — Supplementary material 1 (DOCX 13 kb) [file 12028_2017_472_MOESM1_ESM.docx]

**Appendix A:** Continuous Cerebrovascular Reactivity Index Definitions and Calculation Methods

Autoregulation Indices and Calculation Methods

| **Index** | **Signals Correlated** | **Signal Averaging (sec)** | **Pearson Correlation Coefficient Calculation Window (min)** | **Index Calculation Update Frequency (sec)** |
| --- | --- | --- | --- | --- |
| PRx | ICP and MAP | 10 | 5 | 10 |
| PAx | AMP and MAP | 10 | 5 | 10 |
| RAC | AMP and CPP | 10 | 5 | 10 |
| Mx | FVm and CPP | 10 | 5 | 10 |
| Mx_a | FVm and MAP | 10 | 5 | 10 |
| Sx | FVs and CPP | 10 | 5 | 10 |
| Sx_a | FVs and MAP | 10 | 5 | 10 |
| Dx | FVd and CPP | 10 | 5 | 10 |
| Dx_a | FVd and MAP | 10 | 5 | 10 |
| Lx | LDF-CBF and CPP | 10 | 5 | 10 |
| Lx_a | LDF-CBF and MAP | 10 | 5 | 10 |

*CBF = cerebral blood flow, CPP = cerebral perfusion pressure, FVd = diastolic flow velocity, FVm = mean flow velocity, FVs = systolic flow velocity, ICP = intracranial pressure, LDF = laser Doppler Flowmetry, MAP = mean arterial pressure, min = minute, sec = seconds.*
