# Supplementary material for: ICP Versus Laser Doppler Cerebrovascular Reactivity Indices to Assess Brain Autoregulatory Capacity
Source: Neurocrit Care. 2017 Oct 17;28(2):194–202. doi: 10.1007/s12028-017-0472-x (PMC5948245; doi:10.1007/s12028-017-0472-x)
Supplement: Supplementary file 3 — Supplementary material 3 (DOCX 13 kb) [file 12028_2017_472_MOESM3_ESM.docx]

**Appendix C: Friedman Test – Supplementary Data**

1. Grand Mean Data

Friedman Test Results – Grand Mean Data

| **Autoregulatory Index Group** | **Q Statistic Value** | **p-value** |
| --- | --- | --- |
| *All Indices* | 301.204 | **<0.0001** |
| *ICP Indices* (PRx/PAx/RAC) | 95.690 | **<0.0001** |
| *TCD Indices* (Mx/Mx_a/Sx/Sx_a/Dx/Dx_a) | 159.025 | **<0.0001** |
| *LDF Indices* (Lx/Lx_a) | 5.586 | **0.018** |

AMP = fundamental amplitude of ICP, CPP = cerebral perfusion pressure, df = degrees of freedom, Dx = diastolic flow index (between FVd and CPP), Dx_a = arterial diastolic flow index (between FVd and MAP), FVd = diastolic flow velocity, FVm = mean flow velocity, FVs = systolic flow velocity, ICP = intracranial pressure, Lx = laser Doppler flow index (between LDF-CBF and CPP), Lx_a = arterial laser Doppler flow index (between LDF-CBF and MAP), Mx = mean flow index (between FVm and CPP), Mx_a = arterial mean flow index (between FVm and MAP), PAx = between AMP and MAP, PRx = pressure reactivity index (between ICP and MAP), RAC = between AMP and CPP. *all values in bold type face are those which reached statistical significance (ie. p<0.05)

1. 10 Second by 10 Second data

Friedman Test Results – 10 Second by 10 Second Data

| **Autoregulatory Index Group** | **Q Statistic Value** | **p-value** |
| --- | --- | --- |
| *All Indices* | 40505.705 | **<0.0001** |
| *ICP Indices* (PRx/PAx/RAC) | 14093.360 | **<0.0001** |
| *TCD Indices* (Mx/Mx_a/Sx/Sx_a/Dx/Dx_a) | 23159.026 | **<0.0001** |
| *LDF Indices* (Lx/Lx_a) | 1175.696 | **<0.0001** |

AMP = fundamental amplitude of ICP, CPP = cerebral perfusion pressure, df = degrees of freedom, Dx = diastolic flow index (between FVd and CPP), Dx_a = arterial diastolic flow index (between FVd and MAP), FVd = diastolic flow velocity, FVm = mean flow velocity, FVs = systolic flow velocity, ICP = intracranial pressure, Lx = laser Doppler flow index (between LDF-CBF and CPP), Lx_a = arterial laser Doppler flow index (between LDF-CBF and MAP), Mx = mean flow index (between FVm and CPP), Mx_a = arterial mean flow index (between FVm and MAP), PAx = between AMP and MAP, PRx = pressure reactivity index (between ICP and MAP), RAC = between AMP and CPP. *all values in bold type face are those which reached statistical significance (ie. p<0.05)
