# Supplementary material for: ICP Versus Laser Doppler Cerebrovascular Reactivity Indices to Assess Brain Autoregulatory Capacity
Source: Neurocrit Care. 2017 Oct 17;28(2):194–202. doi: 10.1007/s12028-017-0472-x (PMC5948245; doi:10.1007/s12028-017-0472-x)
Supplement: Supplementary file 4 — Supplementary material 4 (DOCX 189 kb) [file 12028_2017_472_MOESM4_ESM.docx]

**Appendix D: Spearman Principle Component Analysis (PCA) – Supplementary Material**

*** PCA = principle component analysis, F = factor, PC = principle component, F1 = PC1 = principle component #1, F2 = PC2 = principle component #2. PC1 and PC2 are the two components which contribute the largest amount of variance to the entire data set.**

***Biplots of PC1 vs. PC2 display which variables contribute variance to PC1 and PC2. The longer the arm connecting (0,0) to the variable (such as PRx), the larger the contribution of that variable. Similarly, the quadrant on the biplot in which the variable falls correlates to its contribution to a particular PC. The upper left quadrant is primarily PC2; lower left quadrant is neither PC1 or PC2; the upper right quadrant is PC1 and PC2; the lower right quadrant is primarily PC1.**

***Eigenvalue tables display the eigenvalue for each principle component (PC) (also denoted F), with the % variability and cumulative variability for each factor/principle component.**

***Scree plot displays the same information form the eigenvalue table in a histogram format, with each F (or PC) along the x-axis, the eigenvalue along the left side y-axis and the % variability along the right y-axis. Furthermore, the red line on the graph displays the cumulative variability with the addition of each F (or PC) moving from F1 to F18.**

***Factor loading tables display the loading of each variable (ie. PRx, etc.) for each principle component. Loading varies from -1 to +1, with negative values indicating that particular variable is less likely to contribute to the variance in that particular factor (F). Similarly, positive loadings indicate that particular variable likely contribute to the variance in that corresponding factor (F).**

***Contribution % of Variables tables displays the % contribution to the variance of each individual variable for each individual factor (F).**

***Correlation between variables and factor table – denotes the strength of association between various autoregulatory indices and the individual factors**

1. **Grand Mean Data Set**

*Loading Biplot: PC1 vs. PC2*

Spearman Type Principle Component Analysis of Autoregulatory Indices – Biplot of PC1 vs. PC2 (Grand Mean Data)

*
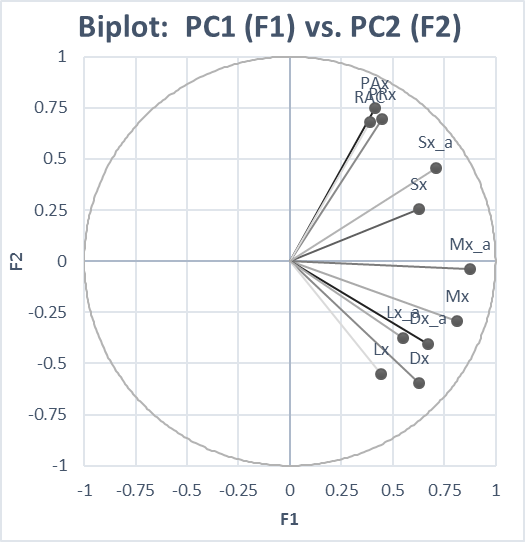
*

PCA = principle component analysis, F = factor, PC = principle component, F1 = PC1 = principle component #1, F2 = PC2 = principle component #2. PC1 and PC2 are the two components which contribute the largest amount of variance to the entire data set. The above biplot of PC1 vs. PC2 displays which variables contribute variance to PC1 and PC2. The longer the arm connecting (0,0) to the variable (such as PRx), the larger the contribution of that variable. Similarly, the quadrant on the biplot in which the variable falls correlates to its contribution to a particular PC. The upper left quadrant is primarily PC2; lower left quadrant is neither PC1 or PC2; the upper right quadrant is PC1 and PC2; the lower right quadrant is primarily PC1.

*Spearman Correlation Matrix*

*Eigenvalue Table*

*Scree Plot*

*
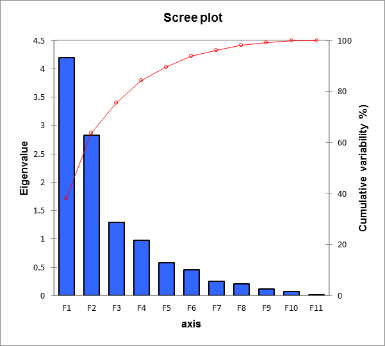
*

*Factor Loading Table*

*% Contribution*

*Correlation Between Variables and Factors*

1. ***10 Second by 10 Second Data***

*Loading Biplot: PC1 vs. PC2*

*
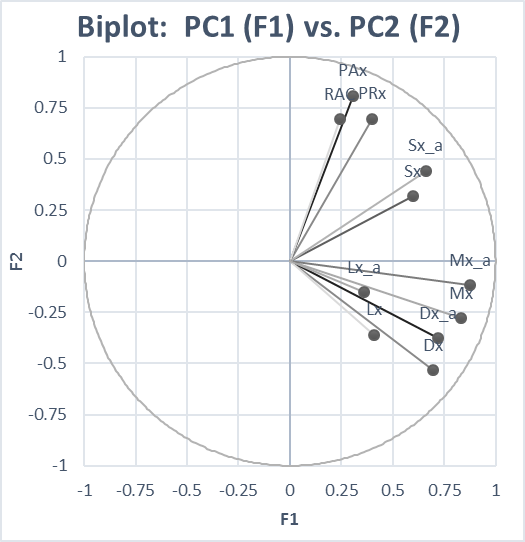
*

*Spearman Correlation Matrix*

*Eigenvalue Table*

*Scree Plot*

*
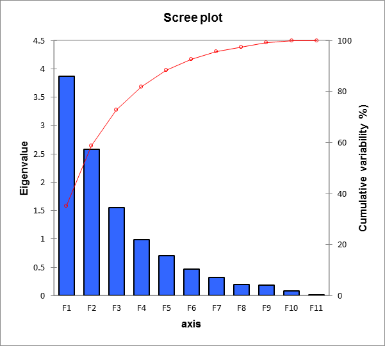
*

*Factor Loading Table*

*% Contribution*

*Correlation Between Variables and Factors*
