# Supplementary material for: ICP Versus Laser Doppler Cerebrovascular Reactivity Indices to Assess Brain Autoregulatory Capacity
Source: Neurocrit Care. 2017 Oct 17;28(2):194–202. doi: 10.1007/s12028-017-0472-x (PMC5948245; doi:10.1007/s12028-017-0472-x)
Supplement: Supplementary file 5 — Supplementary material 5 (DOCX 45 kb) [file 12028_2017_472_MOESM5_ESM.docx]

**Appendix E: Agglomerative Hierarchal Clustering – Supplement**

A: LDF Cohort

1. 10 Second by 10 Second - AHC Dendrogram (Cophenetic correlation = 0.70)

LDF Cohort - AHC Dendrogram – 10 Second by 10 Second Data


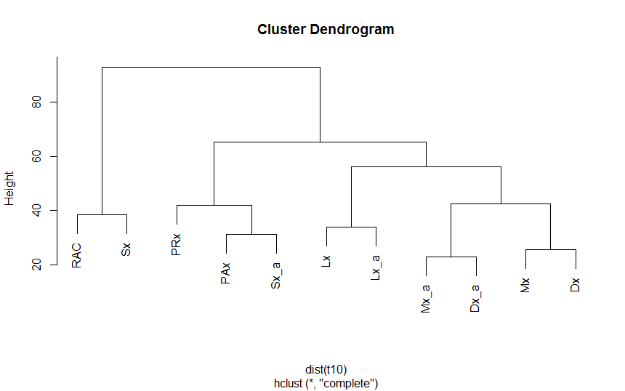


AHC = agglomerative hierarchal clustering, AMP = fundamental amplitude of ICP, CPP = cerebral perfusion pressure, Dx = diastolic flow index (between FVd and CPP), Dx_a = arterial diastolic flow index (between FVd and MAP), FVd = diastolic flow velocity, FVm = mean flow velocity, FVs = systolic flow velocity, ICP = intracranial pressure, Lx = laser Doppler flow index (between LDF-CBF and CPP), Lx_a = arterial laser Doppler flow index (between LDF-CBF and MAP), Mx = mean flow index (between FVm and CPP), Mx_a = arterial mean flow index (between FVm and MAP), PAx = between AMP and MAP, PRx = pressure reactivity index (between ICP and MAP), RAC = between AMP and CPP
