# Supplementary material for: ICP Versus Laser Doppler Cerebrovascular Reactivity Indices to Assess Brain Autoregulatory Capacity
Source: Neurocrit Care. 2017 Oct 17;28(2):194–202. doi: 10.1007/s12028-017-0472-x (PMC5948245; doi:10.1007/s12028-017-0472-x)
Supplement: Supplementary file 6 — Supplementary material 6 (DOCX 94 kb) [file 12028_2017_472_MOESM6_ESM.docx]

**Appendix F: K-Means Cluster Analysis (KMCA) – Supplement**

1. Grand Mean Data

*Clustering Table (k=4)*

| Cluster 1 | Cluster 2 | Cluster 3 | Cluster 4 |
| --- | --- | --- | --- |
| PRx | RAC | Mx | Lx |
| PAx | Sx | Mx_a | Lx_a |
| Sx_a |  | Dx |  |
|  |  | Dx_a |  |

*Within Cluster Sum of Squares (SS):*

*5.344740, 2.247928, 3.139528, 7.737095*

*(between_SS / total_SS = 74.2 %)*

*Elbow Plot:*

*
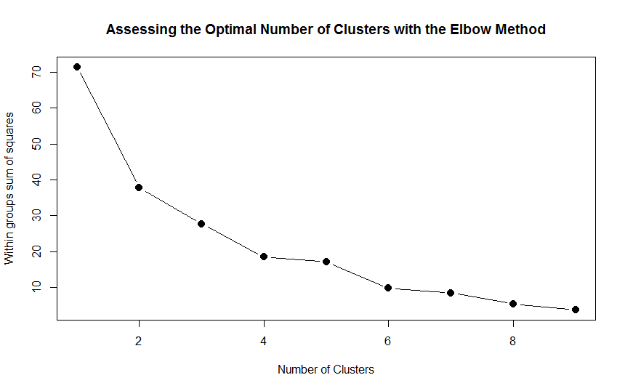
*

1. *10 Second by 10 second Data*

*Cluster Table*

| Cluster 1 | Cluster 2 | Cluster 3 | Cluster 4 |
| --- | --- | --- | --- |
| PRx | RAC | Mx | Lx |
| PAx | Sx | Mx_a | Lx_a |
| Sx_a |  | Dx |  |
|  |  | Dx_a |  |

*Within Cluster Sum of Squares (SS):*

*735.9412, 1678.8637, 1291.3020, 575.5499*

*(between_SS / total_SS = 73.1 %)*

*Elbow Plot:*

*
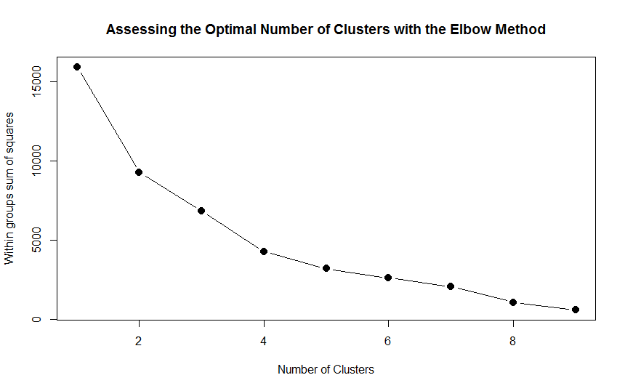
*
